# Evaluation of motion artefact reduction depending on the artefacts’ directions in head MRI using conditional generative adversarial networks

**DOI:** 10.1038/s41598-023-35794-1

**Published:** 2023-05-26

**Authors:** Keisuke Usui, Isao Muro, Syuhei Shibukawa, Masami Goto, Koichi Ogawa, Yasuaki Sakano, Shinsuke Kyogoku, Hiroyuki Daida

**Affiliations:** 1 Department of Radiological Technology, Faculty of Health Science, Juntendo University, Tokyo, Japan; 2 Faculty of Science and Engineering, Hosei University, Tokyo, Japan

**Keywords:** Electrodiagnosis, Medical imaging, Magnetic resonance imaging

## Abstract

Motion artefacts caused by the patient’s body movements affect magnetic resonance imaging (MRI) accuracy. This study aimed to compare and evaluate the accuracy of motion artefacts correction using a conditional generative adversarial network (CGAN) with an autoencoder and U-net models. The training dataset consisted of motion artefacts generated through simulations. Motion artefacts occur in the phase encoding direction, which is set to either the horizontal or vertical direction of the image. To create T2-weighted axial images with simulated motion artefacts, 5500 head images were used in each direction. Of these data, 90% were used for training, while the remainder were used for the evaluation of image quality. Moreover, the validation data used in the model training consisted of 10% of the training dataset. The training data were divided into horizontal and vertical directions of motion artefact appearance, and the effect of combining this data with the training dataset was verified. The resulting corrected images were evaluated using structural image similarity (SSIM) and peak signal-to-noise ratio (PSNR), and the metrics were compared with the images without motion artefacts. The best improvements in the SSIM and PSNR were observed in the consistent condition in the direction of the occurrence of motion artefacts in the training and evaluation datasets. However, SSIM > 0.9 and PSNR > 29 dB were accomplished for the learning model with both image directions. The latter model exhibited the highest robustness for actual patient motion in head MRI images. Moreover, the image quality of the corrected image with the CGAN was the closest to that of the original image, while the improvement rates for SSIM and PSNR were approximately 26% and 7.7%, respectively. The CGAN model demonstrated a high image reproducibility, and the most significant model was the consistent condition of the learning model and the direction of the appearance of motion artefacts.

## Introduction

Patient motion during magnetic resonance imaging (MRI) acquisition may cause image blurring, ringing, and ghosting in the reconstructed image, thereby decreasing the accuracy of the diagnosis^1,2^. Moreover, these image artefacts affect quantitative image data measurements, including anatomical structure volume, borders, and signal value^3^. In addition, motion artefacts induce aliasing along the phase-encoding direction because of the relatively long magnetic resonance (MR) examination time^4^. Therefore, the modulation of data acquisition in the k-space has been strategized, and various motion correction approaches for MR image quality adjustments can be implemented^5^. In prospective motion correction, both parallel imaging methods and online pulse sequence modification synchronized with patient movement reduce perturbations during k-space data acquisition^6–8^. Considering that a single corrupted data in k-space would cause a motion artefact across the entire image, in retrospective motion correction, k-space data were corrected with reference to a priori motion knowledge for modifying the data to a certain extent, including translational motion by applying a linear phase shift^9,10^. In these two approaches for correcting MRI motion artefacts, the order of acquisition of k-space data and the modification of these data were limited to the technique of the echo sequences for each imaging parameter^11^. Moreover, the data assigning method in the k-space with synchronization of the respiratory motion depends on the quality of the patient motion measurement. Therefore, large and irregular motion artefacts may result in the loss of actual image information through motion correction^2,7^.

Owing to recent advancements in deep learning in the medical imaging field, several approaches have been suggested as potential solutions to reduce MRI motion artefacts^11–13^. In this context, Kromrey et al.^11^ investigated the use of a convolutional neutral network (CNN) to reduce respiratory motion artefacts in gadoxetate disodium-enhanced MR images of the liver. Additionally, Duffy et al. compared the CNN performance in two-dimensional (2D) and three-dimensional image approaches and revealed the advantages of signal stability or artefacts created across all three dimensions of data^12,13^. During the training of the CNNs, motion-corrupted images were considered as input data, and motion-free images of the same person were considered as the ground truth. Conversely, acquiring large amounts of coupled data for training deep neural networks is difficult. Therefore, to correct real motion artefact-corrupted images, realistic motion recreation of the original MR image was tested for training CNNs while mixing clean images with motion-simulated data^14,15^. In contrast, some reports have revealed that motion-corrected images using CNN methods are sometimes too smooth and lack visual authenticity, depending on the mean square error (MSE) loss function^16^. Recently, the generative adversarial network (GAN) model has imposed an opposite conversion and achieved high-precision uniformity when underlying similar structures, even when mapping nonlinear fields^17,18^. The adversarial loss calculated using a discriminator formulated in GANs offers a smart training method that imposes advanced-order consistency, which is convenient for motion correction in MR images^19^. Johnson et al. approached the motion correction of a head T2*-weighted image using the GAN model trained with simulated motion artefact images^20^. Moreover, Usman et al.^21^ investigated the efficacy of motion artefact correction for multishot MRI using the GAN model and revealed the robustness of the correction accuracy between the motion magnitude, motion estimation quality, and number of echoes. If these methods aim to be applied clinically at a large number of facilities, superiority compared with previous methods and the dependence on correction accuracy between motion artefact direction and training dataset is important because a deep-learning model requires a lot of learning time using a large amount of training data. Furthermore, this technique should ideally have a more robust model that does not depend on the direction of the motion artefacts. Therefore, the effect of the motion direction in the training data on the motion reduction accuracy must be clarified.

In this study, we investigated the effects of motion artefact reduction using a conditional GAN (CGAN) model in MR images of the head based on several simulated motion artefact images. In addition, motion artefacts in MR images occur in the phase-encoding direction, which can be set in the horizontal and vertical directions of the image. Therefore, to enable the efficient operation and robustness of training data in deep learning, we evaluated the accuracy of motion reduction by comparing and verifying the combination of the direction of motion artefacts and the training data. Furthermore, we compared the effects of the GAN-based model correction method with those of the autoencoder (AE) and U-net models to clarify the image quality and improved effects.

## Methods

### Image data acquisition

The study was approved by the Institutional Review Board (Tokai University Hospital, Japan), and written informed consent was obtained from all participants. This study was conducted in compliance with the principles of the Declaration of Helsinki.

MR images of the head were used to train the deep-learning model and generate simulated motion images. In these datasets, MR images using an Ingenia 1.5T R5.6 (Philips Healthcare, Best, the Netherlands) and a dStream receiver head-spine coil (Philips Healthcare, Best, the Netherlands) were acquired. T2-weighted images with fast spin-echo sequence were acquired using the following parameters: Repetition Time (TR) = 3000 ms, Echo Time = 90 ms, echo train length = 13, field of view = 240 × 240 mm^2^, slice thickness/intersection = 5.0/1.0 mm, matrix size = 256 × 256, and acquisition time = 62 s, and 24 slices per one patient was acquired. The imaging plane was axial. Head images were obtained without movement, and the collected images were obtained from 20 healthy human volunteers with the approval of the Institutional Review Board.

### Generation of simulated motion artefact images

**Figure 1:**
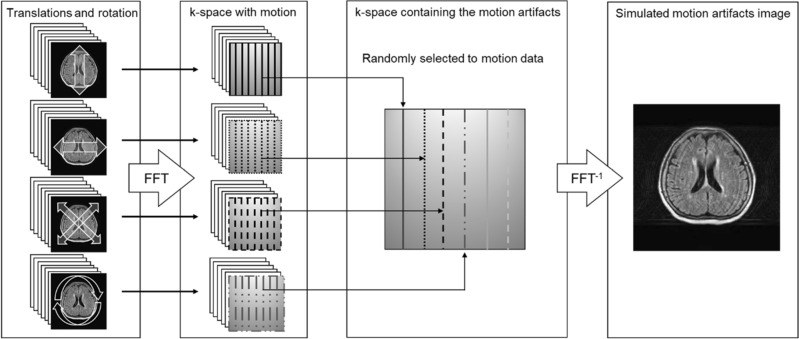
Overview of creating the simulated motion artefacts image in the magnetic resonance images.

Simulated images of the motion artefacts were generated according to our previous method^22^. In this method, the original images were shifted by ± 10 pixels with a one pixel interval in the vertical, horizontal, and diagonal directions. Additionally, rotational images were generated by rotating the original images by ± 5° with a 0.5° interval and using the image center as the rotation center. Therefore, we created 20 types of images with different amounts of pixel shift in the vertical, horizontal, and diagonal directions, as well as 20 types of images with different amounts of rotation. In total we prepared 80 types of movement images, including those in translation and rotation. This motion artefact was simulated only along the 2D in-plane axis. The images were converted to signal intensity with the corresponding frequency using the Fourier transform, and the K-space data were created. Motion artefacts of the head image consists of various types and amounts of motion, including rotation and translation movements, which were combined to generate a head motion artefact image. Subsequently, we randomly selected sequential matrix data from the translated and rotated images. By rearranging the selected data for phase encoding, we created k-space data containing motion artefacts, and used an inverse Fourier transform to generate simulated images with motion artefacts. Therefore, the motion artefact image involved k-space data for both rotational and translational motions. An overview of the process used to create the motion artefact images is shown in Fig. 1. In this study, we simulated motion artefacts in a fast spin-echo sequence and assigned phase-encoding data to each TR. Therefore, assuming that patient motion occurred in each TR, we reordered the encoding data into randomly selected k-space data from the translation and rotation images. We used paired supervised images with 5500 datasets of both the original and simulated images for the training and evaluation of the deep-learning model. In this dataset, we created 5500 motion artefact images in both the horizontal and vertical directions.

### Motion artefacts reduction based on the deep learning model

All datasets contained 5500 T2-weighted images in both horizontal and vertical directions with motion artefacts. Furthermore, 90% of the artefact direction images were used for training, whereas the remainder were used to evaluate the image quality. The validation data for the training model were consistent for 10% of the training datasets. Therefore, we divided the datasets containing motion artefacts into three subsets: training, validation, and testing. These datasets contained 4455, 495, and 550 images, respectively. We created a training model for each of the three motion directions of the artefact images in the horizontal, vertical, and both (horizontal and vertical) directions. In the training model for both artefact directions, there were 2475 datasets with horizontal motion artefacts and 2475 vertical motion artefacts. Therefore, the total number of training datasets for each artefact occurrence direction was the same with a total of 4950. Moreover, we normalized each pixel value to a range of (0, 1) based on the minimum and maximum intensities in the input data and to a range of (0, 255) in the output image. The motion-corrected images were then generated using the CGAN model. Furthermore, the AE and U-net models were applied to generate another set of motion-corrected images for comparison. The details of these deep-learning models are presented below.

#### CGAN

**Figure 2:**
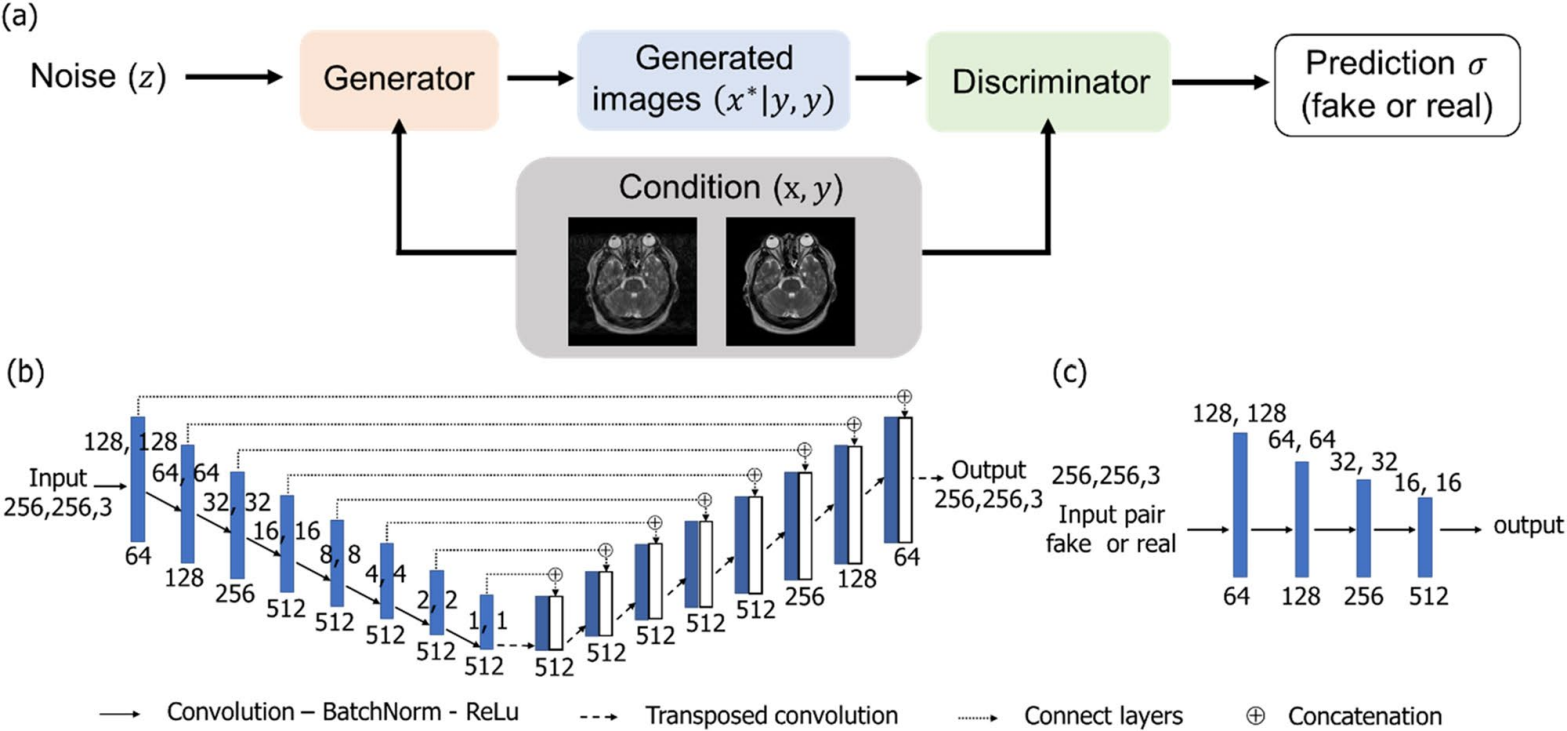
(**a**) Conditional generative adversarial network (CGAN) framework, (**b**) network structure of the generator, and **c** discriminator. The training model consists of one generator and one discriminator with a conditional argument. To train the CGAN, the overall network’s performance is enhanced through networks acting bidirectionally with each other. A motion artefact correction image is generated by a network that maps images from a source domain (with motion artefact) to the target domain (artefacts correction image) based on the conditional ideal image.

A CGAN has two CNNs: a generator and discriminator, each with a given conditional argument that performs opposite functions. During the training process, the generator generates a motion-corrected image from arbitrary noise, whereas the discriminator network attempts to distinguish between real and artificially created images. In this study, conditional mapping was provided to both the generator and discriminator in the CGAN model. The generator attempts to deceive the discriminator and learn the mapping between the generator and target images. Therefore, when the generator images were inputted into the training process, the generator output was similar to that of the target images. Moreover, the discriminator network learns to recognize only real label pairs and provides a probability value that indicates the certainty of matching the label. In this study, the conditional label was defined as an image pair of the original and simulated motion artefact images. Figure 2 presents the workflow of the CGAN model along with the generator and discriminator architectures, which use modules in the form of Convolution-Batch Norm-ReLu. We applied the “U-Net” architecture with skip connections as the generator network, taking the simulated motion artefacts image as input. The skip connection concatenates all the channels in each layer and preserves the local image information that was lost during the down sampling process. Moreover, we utilized the Patch-GAN in the discriminator, which classifies each given patch as either a true or a generated image. Local image patches should be penalized in the discriminator to obtain an accurate model of the high-frequency components. In addition, four convolutional layers were added to the discriminator architecture. The total loss function for this CGAN training is as follows^23^:

1 $$ L_{CGAN} \left( {G, D} \right) = {\mathbb{E}}_{x, y} \left[ {\log D\left( {x, y} \right)} \right] + {\mathbb{E}}_{x, z} \left[ {\log \left( {1 - D\left( {x, G\left( {x, z} \right)} \right)} \right)} \right] $$

In this case, the generator ($$G)$$ attempts to minimize the loss function ($${L}_{CGAN}\left(G, D\right)$$) while the discriminator ($$D$$) strives to maximize it to distinguish between the generated samples $$G\left(x, z\right)$$ and the real samples $$y$$. To ensure effective training, we also included the estimation error loss as feedback to the discriminator. Therefore, the final objective function is as follows:

2 $$ G^{*} = arg\mathop {\min }\limits_{G} \mathop {\max }\limits_{D} L_{CGAN} \left( {G, D} \right) + \lambda L_{{L_{1} }} \left( G \right) $$

here ${L}_{{L}_{1}}\left(G\right)$ is an additional L_1_-norm based on the loss of function in the generator to get closer to the ground truth output, and $\lambda$ is a tunable parameter, for which we set the value to 100. The tests were performed on a personal computer equipped with two GPUs (Quadro RTX 5000, NVIDIA Corporation) and a CPU (Intel Xeon Silver 4210R) with 96 GB of memory. Our algorithm was implemented using MATLAB 2022b (MathWorks Inc., Natick, MA, USA), and the network was optimized using the Adam optimizer with a learning rate of 0.0002 in both the generator and discriminator networks. Additionally, we used a batch size of 1, and training was stopped at 100 epochs. The loss curves demonstrated a continuous decrease as the epoch increased and remained almost unchanged at 100 epochs.

#### The AE model

The AE model had four stack layers, each consisting of an encoder and decoder processes^24^. Each stack contained Convolution-ReLu-MaxPooling modules, which reconstructs the input images and creates same-sized output images. The filter size was 2 × 2, and the number of channels in the first stack was 32. In this model, we used the simulated motion artefact images as input, and the output was the original image without motion artefacts. During the learning process, the network is trained to match the input and output, extract only the important information necessary for image restoration and generate the original data. As a result, the AE can restore a motionless image by extracting the main features representing the original image from the motion-contaminated image and passing this feature through the decoder.

#### The U-net model

The U-Net model has four encoder and decoder depths, and each layer contains Convolution-ReLu-MaxPooling modules. Additionally, skip connections were used in the channels at each layer to restore the overall location information while preserving local features^25^. The upsampled features and their corresponding levels of context aggregation features were recombined using concatenation. The filter size was 3 × 3, and the number of channels in the first layer was 64. This network consists of a context aggregation pathway that gradually encodes the abstract representation of the input and a localization pathway that recombines these representations with shallower features. Therefore, the input images were simulated motion artefact images, whereas the output images were the original images paired with the input images. The optimized network, learning rate, and batch size were identical to those used for the AE model. Training was stopped at 100 epochs, and the loss curves continuously decreased and remained almost unchanged for approximately 100 epochs.

### Image quality evaluation

To evaluate the accuracy of motion correction using the CGAN model, 550 evaluation images that were not included in the training data were corrected using the trained CGAN model. Furthermore, motion reduction was performed using the AE and U-net models to compare image correction quality. This dataset was not included in the training data and did not necessarily contain the same number of motion artefacts as the training images. The quality of the motion artefact-reduced images was quantitatively evaluated by comparing them with the original MR images. To evaluate the qualitative and quantitative differences of the overall images, the SSIM and PSNR values were calculated for the motion corrected images based on the original MR images^26,27^. The SSIM of images $X$ and $Y$ was defined as follows:

3 $$ SSIM\left( {X, Y} \right) = \frac{{\left( {2\mu_{X} \mu_{Y} + C_{1} } \right)\left( {2\sigma_{X} \sigma_{Y} + C_{2} } \right)}}{{\left( {\mu_{X}^{2} + \mu_{Y}^{2} + C_{1} } \right)\left( {\sigma_{X}^{2} + \sigma_{Y}^{2} + C_{2} } \right)}} $$

where ${\mu }_{X}$ and ${\mu }_{Y}$ are the average pixel values of the image pair ($X, Y$), ${\sigma }_{X}$ and ${\sigma }_{Y}$ are the variances, and the $C$ terms are the regularization constants, where ${C}_{1}$ = ${\left(0.01 \times 2000\right)}^{2}$, ${C}_{2}$ = ${\left(0.03 \times 2000\right)}^{2}$, and 255 is the dynamic range of the images. The PSNR was defined as follows:

4 $$ \begin{aligned} & PSNR = 10\log_{10} \frac{{\max \left| {X\left( {i,j} \right)} \right|^{2} }}{MSE} \\ & MSE = \frac{1}{M \times N}\sum\nolimits_{i = 1}^{M} {\sum\nolimits_{j = 1}^{N} {\left( {X\left( {i,j} \right) - Y\left( {i,j} \right)} \right)^{2} } } , \\ \end{aligned} $$

The PSNR is defined as the maximum value in an input image $X\left(i,j\right)$ divided by the MSE between images $X$(with motion artefacts or the corrected image) and $Y$ (the original MRI image). Additionally, $M$ and $N$ represent the widths and heights of the images, respectively. We evaluated the significant differences in the SSIM and PSNR values using a statistical two-tailed t-test; *p* values < 0.005 were considered statistically significant.

### Ethical approval

All procedures were performed in accordance with the ethical standards of the institution or practice at which the study was conducted.

### Informed consent

Informed consent was obtained from all individual participants included in the study.

## Results

**Figure 3:**
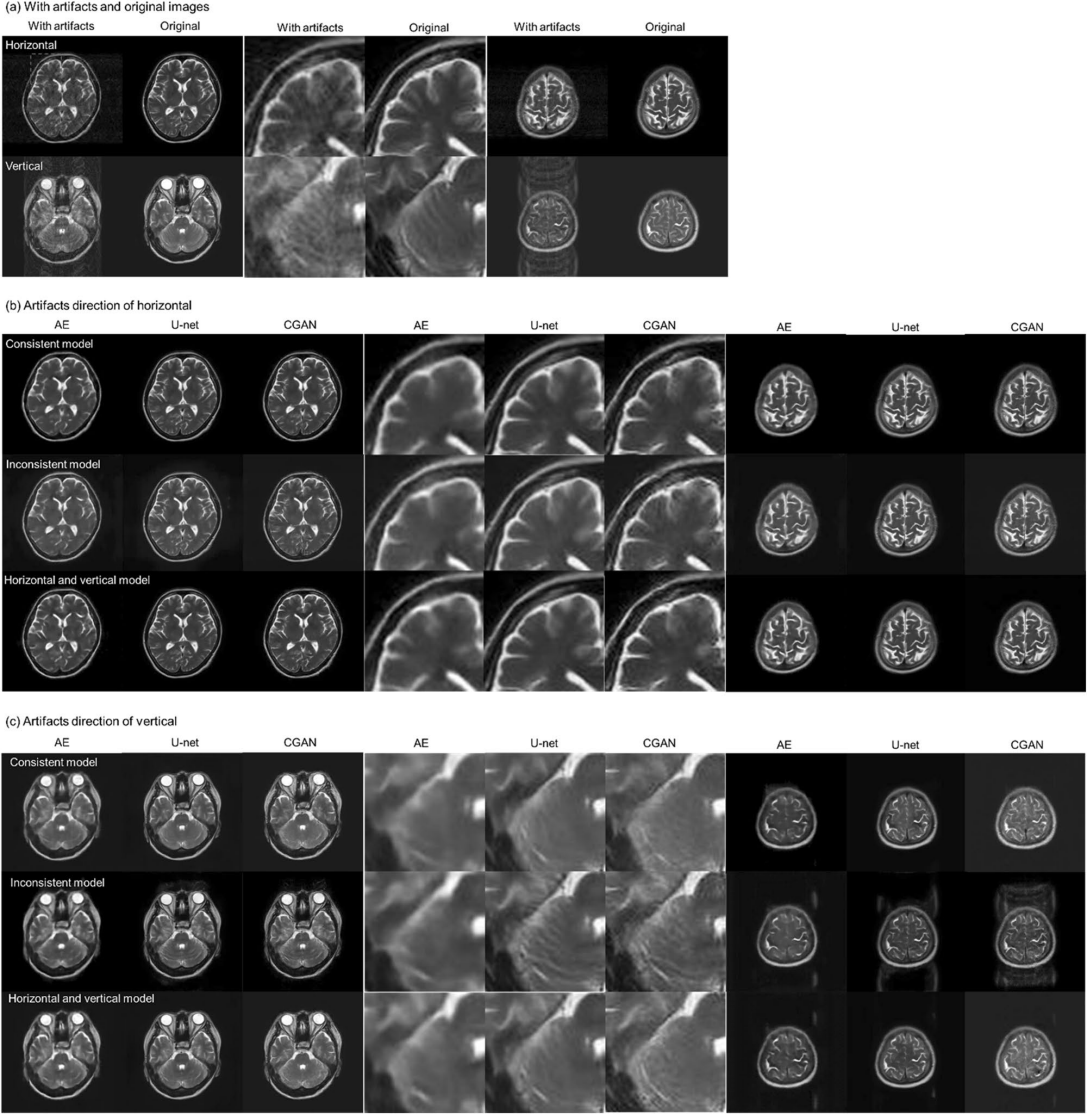
Results of motion-corrected images by the autoencoder (AE), U-net, and conditional generative adversarial network (CGAN) models. (**a**) Simulated motion artefacts images and original magnetic resonance images, (**b**) Motion artefacts occurred in the horizontal direction, and (**c**) in the vertical direction. These models were trained using the image data; consistent with the direction of the motion, inconsistent with the direction of the motion, and with motion artefacts in the horizontal and vertical directions. Enlarged images of the region of interest, indicated in red a, are shown at the center of each image. For example, slices of the parietal image were shown on the right side of each image.

**Figure 4:**
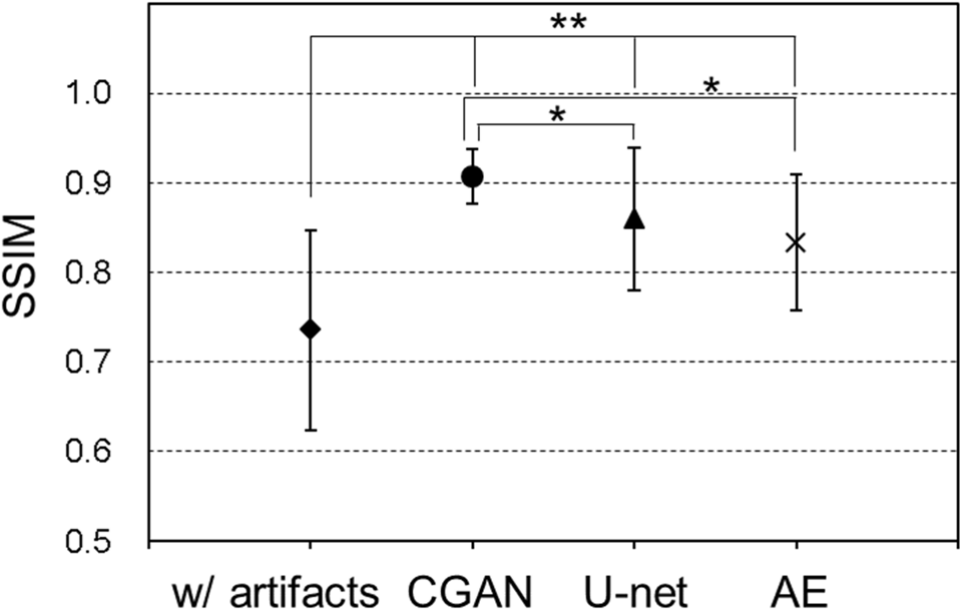
SSIM under training conditions consistent with the direction of the motion artefacts. * indicates *p* < 0.005, showing a significant difference to the result of CGAN, ** indicates *p* < 0.005, showing a significant difference to the results with corresponding motion artefacts image.

**Figure 5:**
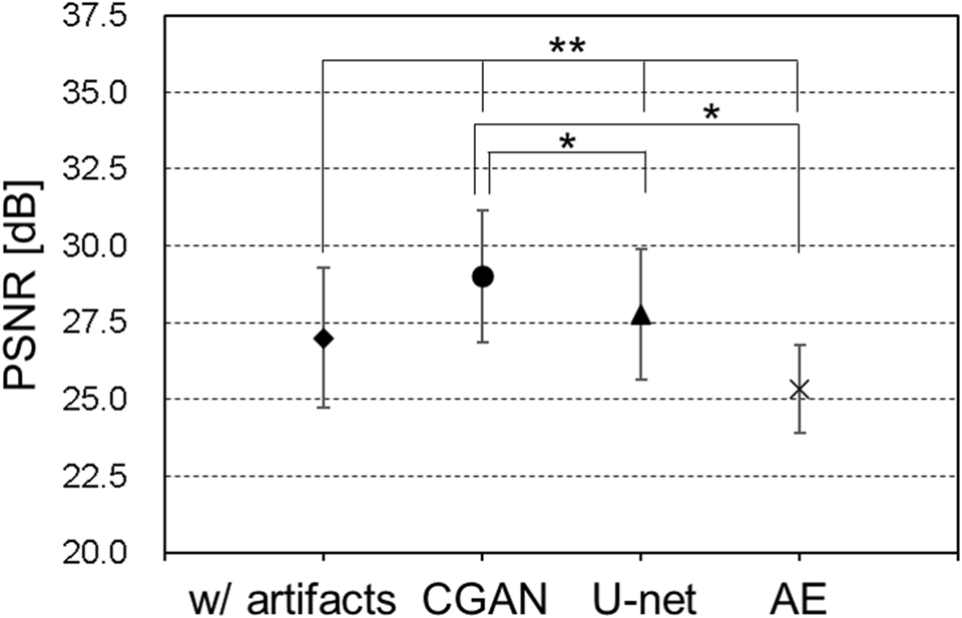
PSNR under training conditions consistent with the direction of the motion artefacts. * indicates *p* < 0.005, showing a significant difference to the result of CGAN, ** indicates *p* < 0.005, showing a significant difference to the results with corresponding motion artefacts image.

Figure 3 shows the results of motion artefact reduction using each deep-learning method in the AE, U-net, and CGAN models: (a) shows an axial slice image of two cases with motion artefacts in the horizontal and vertical directions, with the corresponding original MR images. (b) shows the results of the artefact reduction in the horizontal direction, and (c) shows the results in the vertical direction. For each result, there were three learning models: a consistent condition of directions in the motion artefacts with the verification image and training data, results of the inconsistent condition, and results of the training models using motion artefacts in both the horizontal and vertical directions. In each image, enlarged images of the region of interest (indicated in red in [a]) are shown at the center, and an example slice of the parietal image is shown on the right side. The simulated motion artefact images had a lower quality than the original MR images. These motion artefacts were reduced by image correction using each deep-learning method. However, the image resolution decreased significantly, and blurry images were generated using the AE model. In contrast, we observed that the U-net and CGAN models reduced motion artefacts while maintaining the image resolution. These models can effectively correct the image quality and visuality. Moreover, under conditions where the training data and motion artefacts of the images were not in consistent directions, the images of the motion artefacts remained near the eyeball, and the correction accuracy was insufficient. In the results of the parietal image, residual motion artefacts remained, and the pixel value consistency with the original image was degraded outside the head region. Tables 1 and 2 present the results of the comprehensive image evaluation using the structural image similarity (SSIM) and peak signal-to-noise ratio (PSNR). These results were presented as mean values and standard deviations for the evaluation of the 550 images synthesized using each deep-learning method. In the results of CGAN model, the SSIM mean was 0.925 ± 0.027, and the PSNR mean was 29.46 ± 2.37 dB, in the consistent condition. Therefore, the SSIM and PSNR values in the CGAN model significantly improved under conditions consistent with the direction of the motion artefacts in the training data. In contrast, the SSIM reached approximately 0.9, and the PSNR exceeded 29 dB in the CGAN model with the motion artefact model in both directions. Figures 4 and 5 show a comparison of the SSIM and PSNR values between the deep-learning models and the corresponding motion artefact images. These results were calculated under training conditions consistent with both the horizontal and vertical motion artefacts. After motion correction of the images using the CGAN method, the SSIM and PSNR significantly improved among the three deep-learning methods. Moreover, compared with simulated motion artefact images, the results of SSIM significantly improved by applying each deep-learning correction method; in contrast, PSNRs decreased in the result of the AE model.

**Table 1:** SSIM values for each learning model under training conditions against the direction of the motion.

W/artefacts		Relationship with training data and motion artefacts direction		
		Consistent	Inconsistent	Both directions
0.736 ± 0.111	AE	0.848 ± 0.062	0.307* ± 0.044	0.848 ± 0.054
	U-net	0.897 ± 0.085	0.396* ± 0.047	0.860* ± 0.080
	CGAN	0.925 ± 0.027	0.318* ± 0.056	0.907* ± 0.029

These values are reported as means ± standard deviations. *P* values were calculated using a two-tailed t-test that compared the results of the consistent motion artefact condition between training and evaluation images.

*indicates *p* < 0.005, showing a significant difference to consistent conditions.

**Table 2:** PSNR values for each learning model under training conditions against the direction of the motion.

W/artefacts		Relationship with training data and motion artefacts direction		
		Consistent	Inconsistent	Both directions
27.00 ± 2.29 [dB]	AE	25.78 ± 1.54 [dB]	22.50* ± 1.17 [dB]	25.63 ± 1.43 [dB]
	U-net	29.22 ± 2.26 [dB]	24.00* ± 1.56 [dB]	27.76* ± 2.14 [dB]
	CGAN	29.46 ± 2.37 [dB]	22.10* ± 1.30 [dB]	29.02* ± 2.15 [dB]

These values are reported as means ± standard deviations. P-values were calculated using a two-tailed t-test that compared the results of the consistent motion artefact condition between training and evaluation images.

*indicates *p* < 0.005, showing a significant difference to consistent conditions.

## Discussion

The results of this study showed that motion artefacts in head MR images can be reduced using deep-learning methods. Therefore, the performance of deep learning, along with the CGAN model, which is expected to achieve accurate synthetic image generation, was evaluated in correcting motion artefacts in head MRI images and compared with that of the U-net and AE models.

As shown in Fig. 1a, significant image-quality degradation with simulated motion artefacts occurred in both the horizontal and vertical directions. The results of the AE model showed that the restoration of the decoding process was probably insufficient due to the suppression of the motion artefact region with a relatively high-contrast resolution component. The results of image correction using the deep-learning models in each motion direction in the training image are shown in Fig. 1b–c. The U-net and CGAN models reduced motion artefacts while maintaining image resolution. However, partial over smoothing was observed at the boundary between the white and gray matter in the U-net model results. This outcome showed the same tendency as in previous studies^28,29^. In the training condition with inconsistent directions of motion artefacts in the training and evaluation images, residual motion artefacts were observed, and the correction accuracy was insufficient. In particular, unsuppressed motion artefacts were significant in the parietal images. Tables 1 and 2 show the SSIM and PSNR values for each deep-learning model involving the relationship between the evaluation data and the motion artefact direction. The highest SSIM and PSNR values were observed under consistent conditions of motion artefact direction in the training and evaluation data. Therefore, these are the characteristics of supervised learning and are important factors in preparing training images for the CGAN model. Both direction models were trained using artefact features of the horizontal and vertical occurrence directions, and the artefact reduction accuracy could be affected by other directional features of artefact images. However, SSIM > 0.9 and PSNR > 29 dB were accomplished even for the learning model of the CGAN in both the horizontal and vertical directions. The results of the U-net and CGAN models, comparing these indices with motion images, showed an improvement in SSIM of approximately 24% and 26%, respectively, whereas the PSNR improved by approximately 4.8% and 7.7%, respectively. Consequently, learning models can be constructed for robustness in the phase-encoding direction by training with motion artefact images in both directions. Therefore, motion artefact models in both directions can be used in clinical practice. Training data argumentation, such as image rotation and cropping, may improve the accuracy of motion artefact reduction regardless of the direction of the motion artefacts. In contrast, in the inconsistent conditions, unsuppressed motion artefacts in the outside head region remained significant, and the pixel values deviated from the original MR image, which was remarkable in the parietal image. Therefore, the SSIM results under inconsistent conditions were significantly degraded.

As shown in Figs. 2 and 3, the CGAN model significantly improved the image quality index in terms of SSIM and PSNR compared with the U-net and AE models. By adding the conditions and L_1_ norm regularization, the CGAN model can significantly reduce the motion artefacts and restore the synthetic image close to the original image. Under these constant conditions in model training and image evaluation, pixel value consistency with the original MR image in the air region (outside the head) was superior in the results of CGAN. In contrast, the correction image by U-net degraded the pixel value restorability, particularly with parietal images, and the standard deviation of SSIM and PSNR also increased, deteriorating the results.

In general, collecting several pixel-by-pixel paired MR images with motion artefacts in a clinical setting is difficult. Therefore, in this study, several images with virtual movements were created from static head MR images using computational simulations, and deep learning was performed using these images. A CGAN should add conditional labels using paired images in the process of image generation, where the effectiveness of image quality improvement is expected compared to other deep-learning methods. The reduction in motion artefacts in head MR images can be practical for improving the accuracy of diagnostic imaging, brain region segmentation, and tumor contour delineation in radiotherapy. However, in this study, the images evaluated using deep-learning models were generated using a motion artefact generator, and the correction effect for the motion artefacts, which occurred owing to the actual head movement, was not verified. For the clinical application of motion artefact reduction in head MR images, it is necessary to evaluate the practicality of motion artefact correction using the CGAN model to verify the accuracy of this learning model for motion artefact reduction in actual head MR images.

To suppress motion artefacts in head MR images, a CGAN model was constructed using motion artefact images created through computational simulation as the training data. The correction effects were evaluated and compared with those of other deep-learning models. The CGAN model demonstrated higher image reproducibility than the AE and U-net models. Furthermore, the most significant model can be constructed by making the learning model and appearance direction of the motion artefacts consistent. However, even the learning model using head MR images with motion artefacts in both the horizontal and vertical directions showed a high image quality improvement. Consequently, we constructed a learning model with high robustness to head-movement artefacts in MR images for clinical situations.

## Data Availability

The datasets used and analyzed in the current study are available from the corresponding author upon reasonable request.
